# Synthesis of Poly(L–lactide)–poly(ε–caprolactone)–poly(ethylene glycol) Terpolymer Grafted onto Partially Oxidized Carbon Nanotube Nanocomposites for Drug Delivery

**DOI:** 10.3390/polym16182580

**Published:** 2024-09-12

**Authors:** Karla J. González-Iñiguez, Edgar B. Figueroa-Ochoa, Antonio Martínez-Richa, Leonardo R. Cajero-Zul, Sergio M. Nuño-Donlucas

**Affiliations:** 1 Centro Universitario de Ciencias Exactas e Ingenierías, Universidad de Guadalajara, Guadalajara 44430, Mexico; josefina.gonzalez@academicos.udg.mx; 2 Departamento de Química, Centro Universitario de Ciencias Exactas e Ingenierías, Universidad de Guadalajara, Guadalajara 44430, Mexico; benjamin.figueroa@academicos.udg.mx; 3 Departamento de Química, Universidad de Guanajuato, Guanajuato 36050, Mexico; richa28@msn.com; 4 Departamento de Ingeniería Química, Centro Universitario de Ciencias Exactas e Ingenierías, Universidad de Guadalajara, Guadalajara 44430, Mexico; leocajero88@gmail.com

**Keywords:** nanocomposites, carbon nanotubes, poly(L–lactide), poly(ε–caprolactone), poly(ethylene glycol), methotrexate

## Abstract

Nanocomposites prepared with a terpolymer of poly(L–lactide) (PLLA)–poly(ε–caprolactone) (PCL)–poly(ethylene glycol) (PEG) and partially oxidized carbon nanotubes (CNTs_po_) were synthesized and characterized to evaluate their ability to act as an effective nanocarrier of the anticancer drug methotrexate. The homopolymers of PLLA and PCL were synthesized through ring-opening polymerization (ROP) and characterized through gel permeation chromatography (GPC). The PLLA–PCL–PEG terpolymers were synthesized through a four-step chemical route using oxalyl chloride as a linker agent and analyzed with ^1^H–NMR, ^13^C–NMR, and FTIR spectroscopies. Additionally, the nanocomposites were characterized through FTIR, and X-ray photoelectron spectroscopy (XPS), as well as the differential scanning calorimetry (DSC) technique. XPS analysis revealed that PLLA–PCL–PEG terpolymer chains are grafted onto CNTs_po_. Moreover, evaluations through FTIR and DSC strongly suggest that the PCL-rich domains are preferentially oriented toward CNTs_po_. The release tests exhibited a “burst effect” profile, which was more evident in the terpolymers than in the nanocomposites. Five models were used to assess methotrexate’s in vitro release. For the nanocomposites, the best fit to the experimental data was obtained using the first-order model, whereas the results obtained from the Korsmeyer–Peppas model indicated that Fickian diffusion drives methotrexate’s release.

## 1. Introduction

The design of novel stimuli-responsive drug-delivery systems (DDSs) that improve the efficiency of conventional drug-delivery therapies responds to current demands in the medical field. DDSs have the ability to activate drug release as a response to external stimuli. Different responsive stimuli can trigger drug delivery. Among these stimuli, those due to changes in the chemical environment in an aqueous medium, such as changes in pH or conditions that favor the hydrolysis reaction [1], have gained special attention, as they can be considered as a reference in preparing a polymeric carrier sensitive to the typic processes of living organisms.

Biocompatible polymers can be utilized as smart or external stimuli-responsive systems. For drugs delivered via systemic routes, it is desirable to develop a polymer-based drug carrier that can transport the drug to the target site and start the drug’s delivery under an external stimulus. However, preparing a new chemical polymeric structure that acts as a sensitive carrier to external stimuli implies overcoming a difficult task due to the associated undesired effects because of their interaction with living matter [2]. The goal is to use a polymer that works like a macromolecular shield, protecting the drug in diverse biological environments that can affect the drug’s efficacy [3].

Among the biodegradable and biocompatible polymers that are capable of protecting adsorbed drugs from degradation induced via the enzymes of living bodies, three polymers can be highlighted: (i) polylactide (PLA; this abbreviation is typically used when stereoregularity is not declared), (ii) poly(ε–caprolactone) (PCL), and (iii) poly(ethylene glycol) (PEG) [4].

PLA is a biodegradable biopolymer derived from renewable sources, and it is commercially available, but it has limited mechanical properties and low thermal resistance [5]. This hydrophobic, aliphatic polyester is used in tissue engineering [6] and preparing implants, drug-delivery materials, and surgical sutures for the human body, owing to its hydrolyzable capacity [7]. At an industrial level, PLA is synthesized in bulk through the ring-opening polymerization (ROP) of lactide (LA), which is a cyclic dimer of lactic acid [8]. Due to the chemical structure of LA having two chiral centers, two optical isomers of LA exist: D,D–lactide (D–LA) and L,L–lactide (L–LA). The products of the polymerization of these isomers are named poly(D–lactide) (PDLA) and poly(L–lactide) (PLLA). As a consequence of their optical purity, they have different thermal properties [9].

PCL is a biocompatible, biodegradable, bioresorbable biopolymer; it is easy to synthesize and popular in the biomedical field. However, under physiological conditions, PCL experiments have shown its slow hydrolysis via the cleavage of its ester groups [10]. This thermoplastic polyester is increasingly used to prepare drug-release materials and regenerative medicine implants, as well as to develop new applications in the field of tissue engineering [11]. PCL is compatible with various drugs, such as antipsychotics, antibiotics, or anti-inflammatory agents [10]. Moreover, owing to its slow kinetic degradation and lack of bioactivity [12], it is suitable for the preparation of long-term DDSs [13]. Because of their hydrophobic nature, lipophilic drugs can be homogeneously distributed in their polymeric chains [14], which is a factor that contributes to achieving modulated drug release.

PEG is highly soluble in water, FDA-approved, and biocompatible. This polyether is widely available for preparing novel DDSs, owing to its low toxicity, non-immunogenicity, non-antigenicity, and biological inertness [15,16]. The covalent addition of PEG to one or more molecules, known as PEGylation, is an efficient chemical approach used for drug delivery [17]. Carriers not modified with hydrophilic polymers are susceptible to phagocytosis and the action of the reticuloendothelial system [3]. To prepare a “stealthy” system, PEGylation can be employed to protect a hydrophobic carrier from opsonization.

Amphiphilic block copolymers formed via hydrophobic and hydrophilic segments of biodegradable and biocompatible polymers have gained considerable attention in drug delivery [18]. PLA, PCL, and PEG have been widely used to prepare nanoparticles of segmented copolymers, and their capacity to act as effective nanocarriers that achieve controlled drug delivery has been investigated [19]. Special attention has been paid to the nanocarrier–architecture relationship and its impact on systems that enable stable and safe drug release [20]. Several studies have reported the efficacy of PLA, PCL, and PEG-based segmented copolymers for drug delivery. Thus, the delivery of drugs (such as doxorubicin, an anticancer drug) from the nanoparticles of PCL–PEG copolymers with a complex topology can be optimized by tuning the structure of these copolymers [21]. Poorly water-soluble drugs (such as silibinin, an anticancer agent) have been moderately incorporated into a PEG–PLA–poly(α–benzylcarboxylate–ε–caprolactone) (PBCL) block copolymer to achieve cancer tumor-targeted delivery [22]. Amphiphilic block copolymers can be prepared through two typical routes: (i) via ring-opening copolymerization [23] and (ii) via a linker agent [24]. In this sense, oxalyl chloride has been used as an efficient linker. Its chemical structure has two acyl chloride groups that easily react with hydroxyl groups. Therefore, acyl chloride-capped polymers can be obtained after adding an excess of oxalyl chloride to polymers with terminal hydroxyl groups. After this, other polymers with hydroxyl end groups can react with the acyl chloride end group to obtain a block copolymer [25].

Although copolymers prepared using PLA, PEG, and PCL have relevant advantages for drug delivery, they also have limitations such as an initial “burst release” induced via the high crystallinity of the PCL [26]. The “burst release” phenomenon occurs when a sensitive polymer used as a drug carrier delivers high concentrations of the transported substance after coming into contact with the release medium. After an initial high delivery, a stable release can be achieved [27]. Some positive and negative consequences of “burst release” are known. For example, using modern antimicrobial drugs is desirable to achieve an “impact” dose in the initial stage, in which a high drug concentration is delivered to reduce bacterial growth in the shortest time possible. After this, in the second stage, it is necessary to obtain a gradual release rate [28]. However, to achieve a long-term optimal release, it is necessary to adjust the initial amount of drug released by reducing the “burst release” [29].

Carbon nanotubes (CNTs) are emerging DDSs suitable for cancer treatment [30]. Owing to their large surface and cavity structures, CNTs can load high amounts of drugs [31]. Although they have outstanding properties, pristine CNTs are cytotoxic; to overcome this drawback, it is necessary to achieve adequate chemical functionalization [32]. Functionalized CNTs (f–CNTs) show suitable biocompatibility, and living organisms can excrete them [33]. Furthermore, f–CNTs have the potential for use in the synthesis of polymer nanocomposites that can serve as nanocarriers. For this, the “grafting to” approach (in which the f–CNTs are attached to a well-defined polymer previously presynthesized [34]) is suitable for the preparation of a polymeric nanocarrier with excellent physicochemical properties. In fact, CNTs are a valuable alternative to other nanostructures, such as boron nitride nanotubes (BNNTs), which also have high potential for use in the preparation of polymer-based nanocarriers [35].

Here, the synthesis and characterization of nanocomposites prepared with f–CNTs obtained from partially oxidized CNTs (in which hydroxyl and carboxyl groups were inserted) and a terpolymer of PLLA, PCL, and PEG, prepared using oxalyl chloride as a linker agent, are reported. These nanocomposites were synthesized to investigate their capacity to act as sensitive nanocarriers. Accordingly, a series of PLLAs and PCLs were synthesized through ROP and characterized using gel permeation chromatography (GPC). Pure PEG and the synthesized PLLAs and PCLs were studied using differential scanning calorimetry (DSC). The PLLA–PCL copolymers and PLLA–PCL–PEG terpolymers were synthesized using oxalyl chloride as a linker agent and characterized through the following spectroscopic techniques: Fourier-transform infrared (FTIR), proton nuclear magnetic resonance (^1^H–NMR), and ^13^carbon nuclear magnetic resonance (^13^C–NMR), as well as DSC. The PLLA–PCL–PEG terpolymers and their nanocomposites were analyzed using X-ray photoelectron spectroscopy (XPS). The characterization of the nanocomposites was completed through FTIR and DSC. The capacity of releasing methotrexate (an anticancer drug) from the PLLA–PCL–PEG terpolymers and their nanocomposites was evaluated through ultraviolet–visible spectroscopy (UV–vis), and various mathematical models were used to fit the experimental data. Moreover, the ability of the nanocomposites to reduce the “burst release” phenomenon detected on the release tests of the pure PLLA–PCL–PEG terpolymers was assessed.

## 2. Materials and Methods

### 2.1. Materials

L–Lactide (L–LA) ((3*S*)–cis–3,6–dimethyl–1,4–dioxane–2,5–dione) (98%), ε–caprolactone (ε–CL) (97%), tin(II) 2–ethylhexanoate (stannous octoate, SnOct_2_) (96%), 1,4–butanediol (BD) (99%), calcium hydride (>90%), triethylamine (ET_3_N) (99.5%), toluene (ACS reagent) (99.5%), methanol (ACS reagent) (99.8%), dichloromethane anhydrous (99.8%), oxalyl chloride (OxCl) (98%), tetrahydrofuran (THF) (ACS grade), poly(ethylene glycol) (PEG) (M_n_ = 10,000), phosphate-buffered saline (PBS) at pH 7.4, methotrexate (4–amino–10–methylfolic acid) MW = 454.44 g/mol, and ethanol (ACS reagent) were purchased from Sigma–Aldrich (Saint Louis, MO). Alumina boats were obtained from Alfa Aesar (Tewksbury, MA). Ethanol absolute (99.5%), hydrochloric acid (ACS reagent), and ferric nitrate nonahydrate (Fe(NO_3_)_3_∙9H_2_O) were acquired from Golden Bell (Guadalajara, Mexico). Petroleum ether (ACS reagent) (distillation range: 20 °C (293.15 K)–60 °C (333.15 K)) was purchased from Almacen de Drogas la Paz (Guadalajara, Mexico). Nitrogen gas (99.99%) was obtained from INFRA (Guadalajara, Mexico). Argon gas (99.998%) was provided by Linde (Guadalajara, Mexico). Deionized and bi-distilled water was purchased from Productos Selectropura (Guadalajara, Mexico). The chemical reagents mentioned above were used without purification, except for ε–CL, which was dried with calcium hydride under constant stirring for 48 h before use. The flask in which the mixture of ε–CL/calcium hydride was deposited was connected to a Schlenk line under a nitrogen atmosphere. Then, an exhaustive refilling process was repeated three times to separate the wet calcium hydride from the dry ε–CL. The dry ε–CL was deposited into a container and placed in a desiccator until use. The amount of water contained in unpurified BD was determined using the Karl Fischer method, resulting in 16.3 wt.%. The measurement was repeated three times. Therefore, the BD used in this work was named wet BD.

### 2.2. Preparation, Purification, and Chemical Modification of the Prepared CNTs

The CNTs were synthesized using the chemical vapor deposition (CVD) method with Fe as a catalyst and ethanol as a carbon source. A detailed description of the synthesis procedure was previously reported by our research group elsewhere [36]. The prepared CNTs were purified using a solution of HNO_3_ (37.5 wt.%) in water (1:3 *v*/*v*). The purified CNTs were repeatedly washed with bi-distilled water until a pH of 7 was achieved and then oven-dried at 50 °C (323.15 K). The detailed purification pathway was previously reported elsewhere [37]. The purified CNTs were named CNTs_puri_. CNTs_puri_ were chemically functionalized through an oxidation process. For this, 1 g of CNTs_puri_ was deposited into a 250-mL glass container of Soxhlet extraction equipment. Subsequently, 140 mL of 4 M nitric acid solution was aggregated. The mixture was heated until the boiling point was reached and maintained under reflux for 5 h. The product (partially oxidized CNTs) was named CNTs_po_, and it was recovered through centrifugation. Finally, the CNTs_po_ were washed with bi-distilled water several times until a pH of 7 was obtained and then oven-dried at 50 °C (323.15 K) for 72 h. The dry CNTs_po_ were stored in a desiccator until use. For this way, carbonyl, hydroxyl, and carboxyl chemical groups were inserted on the walls of the CNTs_po_.

### 2.3. Synthesis of PLLA–PCL–PEG Terpolymers

**Scheme 1:**
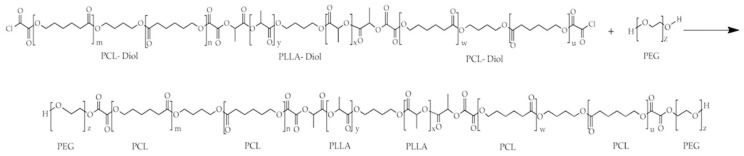
Expected chemical reaction for the preparation of PLLA–PCL–PEG terpolymers.

PLLA–PCL–PEG terpolymers were synthesized following a chemical path of four steps. First, a PLLA homopolymer was synthesized through ROP using wet BD as an initiator and SnOct_2_ as a catalyst. The polymerization reaction was carried out in a 150-mL two-neck, round-bottom glass flask previously dried at 60 °C (333.15 K) for 24 h. L–LA (15 g), wet BD, and SnOct_2_ at specific amounts, as well as a stirring bar (previously dried at 50 °C (323.15 K) for 24 h), were placed into the flask and connected to a reflux condenser. The flask was partially submerged in an oil bath maintained at 110 °C (383.15 K). Polymerization was performed under a nitrogen atmosphere with stirring for 1 h. After the reaction was completed, the flask was cooled to room temperature. To purify the prepared product, it was dissolved in 20 mL of dichloromethane and poured dropwise into 60 mL of petroleum ether to induce precipitation. The product was maintained at room temperature for 3 h, and the precipitate was recovered through centrifugation. Subsequently, it was washed with methanol and dried at 45 °C (318.15 K) until a constant weight was reached. Four lineal PLLAs were synthesized at the same molar ratio of [L–LA]/[SnOct_2_] = 300/1 but at four different molar ratios of [L–LA]/[wet BD], which were named as follows: (i) PLLA 1, obtained at [L–LA]/[wet BD] = 30/1; (ii) PLLA 2, prepared at [L–LA]/[wet BD] = 40/1; (iii) PLLA 3, synthesized at [L–LA]/[wet BD] = 50/1; and (iv) PLLA 4, manufactured at [L–LA]/[wet BD] = 60/1. The expected product of this synthesis is PLLA–diol. Second, a series of PCLs were synthesized through ROP again. For this, 10 g of dry ε–CL and a dry stirring bar were placed in a previously dried, 150-mL two-neck, round-bottom glass flask. Then, specific amounts of wet BD and SnOct_2_, as well as a dry stirring bar, were added, and the flask was partially submerged in an oil bath at 110 °C (383.15 K). The reaction was carried out, maintaining constant nitrogen bubbling with stirring for 1 h. After the reaction was completed, the flask was cooled to room temperature. The reaction product was purified by dissolving it in 20 mL of dichloromethane and then precipitated by adding 60 mL of petroleum ether. The prepared PCL was recovered through centrifugation. Thereafter, the prepared PCL was oven-dried at 45 °C (318.15 K) until a constant weight was reached. Four lineal PCLs were prepared, maintaining the same molar ratio [ε–CL]/[SnOct_2_] = 300/1 but different molar ratios of [ε–CL]/[wet BD]. The synthesized PCLs were named PCL 1, PCL 2, PCL 3, and PCL 4, and they were synthesized at [ε–CL]/[wet BD] = 30/1, 40/1, 50/1, and 60/1, respectively. Following this procedure, PCL diol species were synthesized. Third, the linear segmented copolymers of PLLA–PCL were synthesized using OxCl as a linker agent, following a two-stage procedure. The four prepared PCLs and the PLLA 1 were chosen to synthesize the PLLA–PCL copolymers. In the first stage, a sample of PLLA 1 reacted with OxCl to prepare a prepolymer. The synthesis was performed in a two-neck, round-bottom glass flask with a septum inlet of 50 mL previously dried at 50 °C (323.15 K) for 12 h. One neck was connected to a reflux condenser, and on the other neck, a septum was adjusted. A steel cannula was inserted into the septum to bubble nitrogen. A certain mass of PLLA 1 was dissolved in 10 mL of dichloromethane, and the dissolution was placed in the flask, which was partially submerged in an oil bath at 35 °C (308.15 K). Then, 30 μL of ET_3_N dissolved in 5 mL of dichloromethane was added to this flask. Separately, 70 μL of OxCl dissolved in 5 mL of dichloromethane was added dropwise. The mixture was maintained under continuous agitation and with nitrogen bubbling for 1 h. Under these conditions, a chemical reaction between the hydroxyl groups of PLLA 1 and chloride acyl groups of OxCl could be carried out to obtain a prepolymer. In the second step, the synthesis of PLLA–PCL copolymers was carried out. For this, immediately after finishing the first stage, a certain mass of one PCL dissolved in 5 mL of dichloromethane was added to the mass of the prepolymer, and the copolymerization reaction started. The reaction was carried out for 1 h. The masses of PLLA 1 and the PCL used were calculated, maintaining a ratio of two moles of the end groups of PCL to one mole of the end groups of PLLA 1. After the reaction time had elapsed, the reaction mixture was cooled to room temperature, allowed to stand for 24 h, and then purified. For this, a mixture of 15 mL of dichloromethane and 15 mL of toluene was added to dissolve the PCL that did not react. The mixture was stirred for 15 min, filtrated, and then oven-dried at 45 °C (318.15 K) until a constant weight was achieved. Subsequently, the dry solid was dissolved in 15 mL of dichloromethane and precipitated with the addition of 45 mL of petroleum ether. The solid obtained (purified PLLA–PCL copolymer) was recovered and oven-dried until a constant weight was achieved. Four PLLA–PCL copolymers were synthesized: (i) PLLA–PCL 1, prepared with PLLA 1 and PCL 1; (ii) PLLA–PCL 2, obtained with PLLA 1 and PCL 2; (iii) PLLA–PCL 3, manufactured with PLLA 1 and PCL 3; and (iv) PLLA–PCL 4, synthesized with PLLA 1 and PCL 4. Fourth, the PLLA–PCL–PEG terpolymers were obtained through a chemical reaction between the PLLA–PCL copolymers and PEG. Again, a chemical route of two stages was followed. OxCl was used as a linker agent. In the first stage, a certain mass of the PLLA–PCL copolymer was treated with OxCl to synthesize a precursor of the terpolymer. To perform the synthesis, a 50-mL two-neck, round-bottom glass flask immersed in an oil bath at 35 °C (308.15 K) was used. Initially, a certain mass of one PLLA–PCL copolymer was dissolved in 10 mL of dichloromethane, and the dissolution was deposited in the flask. Then, 30 μL of ET_3_N dissolved in 5 mL of dichloromethane was added. Another solution of 70 μL of OxCl dissolved in 5 mL of dichloromethane was added. The mixture was reacted for 1 h under a nitrogen atmosphere. In this way, the hydroxyl end groups of the PLLA–PCL copolymer can react with one acyl chloride group of OxCl. In the second stage, the complete preparation of the terpolymers was achieved. After the reaction time of the first stage had elapsed, a certain mass of PEG dissolved in 5 mL of dichloromethane was added to the precursor, and the mixture was maintained under nitrogen bubbling for 1 h. The mass of PEG added was calculated to maintain a molar ratio of two moles of the end groups of PEG by one mole of the end groups of the PLLA–PCL copolymer, under the assumption that this copolymer has a chemical structure composed of three segments: PCL–PLLA–PCL. The reaction product was cooled to room temperature and then purified after 24 h. Purification was carried out by washing the product prepared with methanol to dissolve the PEG that did not react. Then, the purified product (PLLA–PCL–PEG terpolymer) was oven-dried at 45 °C (318.15 K) until constant weight was reached. The prepared terpolymers were named PLLA–PCL–PEG 1, PLLA–PCL–PEG 2, PLLA–PCL–PEG 3, and PLLA–PCL–PEG 4, which were synthesized via the chemical reaction between PLLA–PCL 1, PLLA–PCL 2, PLLA–PCL 3, and PLLA–PCL 4 with PEG, respectively. Scheme 1 shows the expected chemical reaction for the preparation of PLLA–PCL–PEG terpolymers from the PLLA–PCL copolymer, as described above.

### 2.4. Synthesis of PLLA–PCL–PEG/CNT_po_ Nanocomposites

To prepare the PLLA–PCL–PEG/CNT_po_ nanocomposites, a certain mass of each PLLA–PCL–PEG terpolymer was dissolved in THF. In this solution, 1.0 wt.% or 0.5 wt.% of CNTs_po_ was added. The dispersion obtained was placed in a Petri dish and sonicated in model 3510R–MTH Bransonic Ultrasonic Cleaner (Danbury, USA) (using an amplitude of 42 KHz ± 6%, and a power of 100 W) for at least 30 min at 40 °C (313.15 K) until the solvent evaporated. The Petri dish was heated in an oven at 45 °C (318.15 K) to eliminate the solvent residual. Subsequently, the prepared and dried nanocomposites were stored in a desiccator. Table 1 lists the names of the PLLA–PCL–PEG terpolymers used as polymer matrices of the prepared PLLA–PCL–PEG/CNT_po_ nanocomposites and the contents (in wt.%) of CNTs_po_ of each nanocomposite.

### 2.5. Characterization of CNTs_po_, PLLA–PCL–PEG Terpolymers, and PLLA–PCL–PEG/CNT_po_ Nanocomposites

The prepared CNTs_po_, synthesized PLLA and PCL homopolymers, homopolymer PEG, synthesized PLLA–PCL copolymers, and PLLA–PCL–PEG terpolymers, as well as the obtained PLLA–PCL–PEG/CNT_po_ nanocomposites, were characterized using the techniques described as follows.

#### 2.5.1. Characterization of the Functionalized CNTs

The amount of carboxyl and hydroxyl groups inserted on the walls of the CNTs_po_ was determined using two methods reported elsewhere [36,38]. Both methods were based on the back-titration of a hydrochloric acid solution. This solution reacts with the excess of a sodium hydroxide solution to determine the amount of hydroxyl groups, or with an excess of a sodium bicarbonate solution to calculate the amount of carboxyl groups. The determinations were conducted based on the mass of CNTs_po_.

#### 2.5.2. GPC

GPC was employed to determine the molar masses of the synthesized polymers and their molar mass distributions. For this, dilute solutions of each sample were prepared. These solutions were prepared by dissolving 10 mg of the sample in 2 mL of THF. Subsequently, they were filtered through a 0.45-mm-pore and 13-mm-diameter filter (GHP Acrodisc Gelman (St. Louis, MO, USA)). The measurements were made in a Waters model 2414 chromatograph provided with Waters Styragel columns, which cover the range of 500–300,000 Da, and a refraction index detector. All tests were conducted using THF as an eluent (1.0 mL/min) at 30 °C (303.15 K). Calibration was performed using polystyrene standards. The data treatment was performed using the Empower software (version 2).

#### 2.5.3. Proton and Carbon–13 Nuclear Magnetic Resonance (^1^H– and ^13^C–NMR)

The ^1^H– and ^13^C–NMR spectra of the PLLA–PCL copolymers and the PLLA–PCL–PEG terpolymers were recorded at room temperature using Bruker Avance III 500 (MHz) (Billerica, USA). Chemical shifts (ppm) were relative to the remaining nondeuterated chloroform signal (from CDCl_3_) and used as an internal reference for ^1^H NMR spectroscopy. The peaks for PLLA moiety appeared at 1.60 and 5.2 ppm due to the (–C**H_3_**) and (–C**H**–) groups, respectively, in all samples. The peaks for PCL appeared at 4.0 ppm [–(CH_2_)_4_C**H_2_**OC(O)–]_n_, 2.3 ppm (–(CH2)_4_C**H2**COO–), 1.6 ppm (–(CH2C**H_2_**CH_2_C**H_2_**CH**2**COO–), and 1.35 ppm (–CH2CH2C**H2**CH2CH2COO–). The end groups due to the PLLA methine end group (–CH_3_C**H**OH) were seen at 4.4 ppm. This peak was superimposed with other peaks. The peaks for the ε–CL end group –C**H_2_**OH appeared at ca. 3.65 ppm, whereas those for PEG methylene (OC**H**_2_CH_2_) appeared at ca. 3.60 ppm.

The ^13^C–NMR spectra were also recorded using a Bruker Avance III 500 MHz spectrometer (Billerica, USA). The peaks for PLLA moiety appeared at 16.7, 69.1, and 169.6 ppm due to the (–**C**H_3_), (–**C**H–), and (**C**=O) groups, respectively, in all samples. The peaks for PCL appeared at 64.2 ppm [–(CH_2_)_4_**C**H_2_OC(O)–]_n_, 34.1 ppm (–(CH2)_4_**C**H2COO–), 28.3 ppm (–(CH2**C**H_2_CH_2_CH_2_CH**2**COO–), 24.5 ppm (–(CH2CH_2_CH_2_**C**H_2_CH**2**COO–), 25.5 ppm (–CH2CH2**C**H2CH2CH2COO–), and 173.6 ppm for ester carbonyl. The signal peak for PEG methylene (O**C**H_2_CH_2_) was seen at 70.5 ppm.

#### 2.5.4. Fourier-Transform Infrared Spectroscopy (FTIR)

The vibrational behaviors of the CNT_po_, PLLA, PCL, and PEG homopolymers, PLLA–PCL–PEG terpolymers, and PLLA–PCL–PEG/CNT_po_ nanocomposites were analyzed using FTIR. For this, a Perkin Elmer Spectrum One spectrophotometer was used. To obtain the spectra, all samples were dried in a vacuum oven at 50 °C (323.15 K) until a constant weight was achieved. Similarly, KBr was dried in a vacuum oven at 100 °C (373.15 K) for 24 h. Then, dry samples were mixed with dry KBr (1 mg of sample by 200 mg of KBr), and pellets were obtained via compression. The spectra were recorded from 4000 to 450 cm^−1^, averaging 50 scans to reduce the signal/noise ratio, and they were obtained at a resolution of 2 cm^−1^.

#### 2.5.5. XPS

The PLLA–PCL–PEG terpolymers and PLLA–PCL–PEG/CNT_po_ nanocomposites were investigated through XPS. The analysis was conducted in a system formed via an XR 50M monochromatic Al *Kα*_1_ (*hν* = 1468.7 eV) X-ray source and a Phoibos 150 spectrometer, which was equipped with a 1D–DLD one-dimensional detector provided by SPECS (Berlin, Germany). For the measurements, the samples were mounted onto a steel sample holder with double-sided copper tape to avoid interference from carbon tape, and they were then dried in a vacuum oven for 24 h. Then, the samples were placed into the prechamber. The measurements were recorded at a base pressure of 4.2 ×10^−10^ mbar, an electron takeoff angle of 90° at 150 W, a pass energy of 10 eV, and a step size of 0.1 eV. A flood gun was used to compensate for the charge on the samples. The recorded XPS spectra were shifted using the reference of the C–C-binding energy position.

#### 2.5.6. DSC

Thermal characterization of the PLLA, PCL, and PEG homopolymers, PLLA–PCL–PEG terpolymers, and PLLA–PCL–PEG/CNT_po_ nanocomposites was performed using a Q–100 calorimeter (TA Instruments). DSC thermograms were obtained with a dynamic heating program in a temperature range of −70 °C (203.15 K) to 200 °C (473.15 K) following a heating rate of 10 °C (10 K)/min. An inert atmosphere was maintained by feeding nitrogen at a flow rate of 50 mL/min to the sample camera. The masses of the samples were in the range of 5 to 5.5 mg. Aluminum pans were used to encapsulate the samples. Two scans were recorded, and the second scan was reported. The glass-transition temperature (T_g_) of all the analyzed materials was determined using the mid-point criteria.

#### 2.5.7. Ultraviolet–Visible (UV–vis) Spectroscopy

UV–vis spectroscopy was employed to assess the in vitro methotrexate release from the tablets of the PLLA–PCL–PEG 2 and PLLA–PCL–PEG 4 terpolymers, as well as the NC 1, NC 2, NC 3, and NC 4 nanocomposites. For this, an Evolution 220 spectrometer (Thermo Fisher Scientific, Waltham, MA, USA) was utilized. The tablets were prepared by compression-mixing a certain mass of the terpolymers or the mentioned nanocomposites with the methotrexate. The total masses of the tablets were in the range of 50 to 85 mg. The mass ratio of terpolymer or nanocomposite to methotrexate was 10/1. The obtained tablets had a thickness of 4 mm. Each prepared tablet was introduced in a dialysis membrane (SpectraPor, MWCO 3500 Da) and deposited in a 500-mL beaker. After this, 200 mL of PBS buffer at pH 7.4 was added. Then, 4 mL of ethanol was added to inhibit the formation of aggregates of the drug methotrexate [39]. The obtained mixture deposited in the beaker was placed in a dark chamber to protect the methotrexate (a photosensitive substance) from visible light at 37 °C (310.15 K). The mixture was maintained under constant stirring at 250 rpm. Samples (1 mL) of this mixture were taken in the range of 0 to 96 h. After each volumetric sample was withdrawn, the same volume of PBS buffer was added. The evolution of methotrexate release was evaluated by determining the methotrexate concentration and evaluating the absorbance at 302 nm as a function of the time using a calibration curve prepared with solutions of methotrexate in PBS. Each release test was repeated twice.

## 3. Results and Discussion

### 3.1. Characterization of the Prepared and Functionalized CNTs

Due to the methodology used to prepare the CNTs in this study being the same as the one already reported by our research group in previous works, some physical characteristics of these nanostructures are known. In this sense, an analysis via high-resolution transmission electron microscopy (HR–TEM) demonstrated that the synthesized CNTs were preferentially multi-walled carbon nanotubes (MWCNTs). Their outer tube diameters ranged from 10 to 45 nm, the graphene sheet numbers ranged from 10 to 40, and the spacing between sheets was ca. 0.34 nm, as was reported elsewhere [40]. Moreover, an analysis using high-resolution scanning electron microscopy (HR–SEM) showed that the population of the purified CNTs (CNTs_puri_) consists of both straight cylindrical CNTs and helically coiled CNTs, as was reported previously [36].

Concerning functionalized CNTs, the quantification of the hydroxyl and carboxyl groups inserted on the walls of the CNTs_po_ was performed using the aforementioned experimental methods. The carboxyl groups represent 33 wt.% per gram of CNTs_po_, whereas the hydroxyl groups represent 15 wt.% per gram of CNTs_po_.

### 3.2. Characterization via GPC of the Homopolymers of PLLA and PCL

The GPC results of the homopolymerization of L–LA and ε–CL and the yield of both polymerizations are listed in Table 2. These results were obtained from the GPC patterns of the synthesized PLLA and PCL. A high yield (≥90%) of all homopolymerization reactions was obtained. As expected, the weight–average molar mass (M_w_) of both linear PLLAs and PCLs increased as the molar ratio [L–LA]/[wet BD] and [ε–CL]/[wet BD] reached higher values. The polydispersity index (PDI = M_w_/M_n_) of the synthesized PLLAs had values close to 1.0, whereas the PDI of the prepared PCLs had slightly larger values.

### 3.3. Analysis of the Chemical Structure of the PLLA–PCL Copolymers, PLLA–PCL–PEG Terpolymers, and PLLA–PCL–PEG/CNT_po_ Nanocomposites

**Figure 1:**
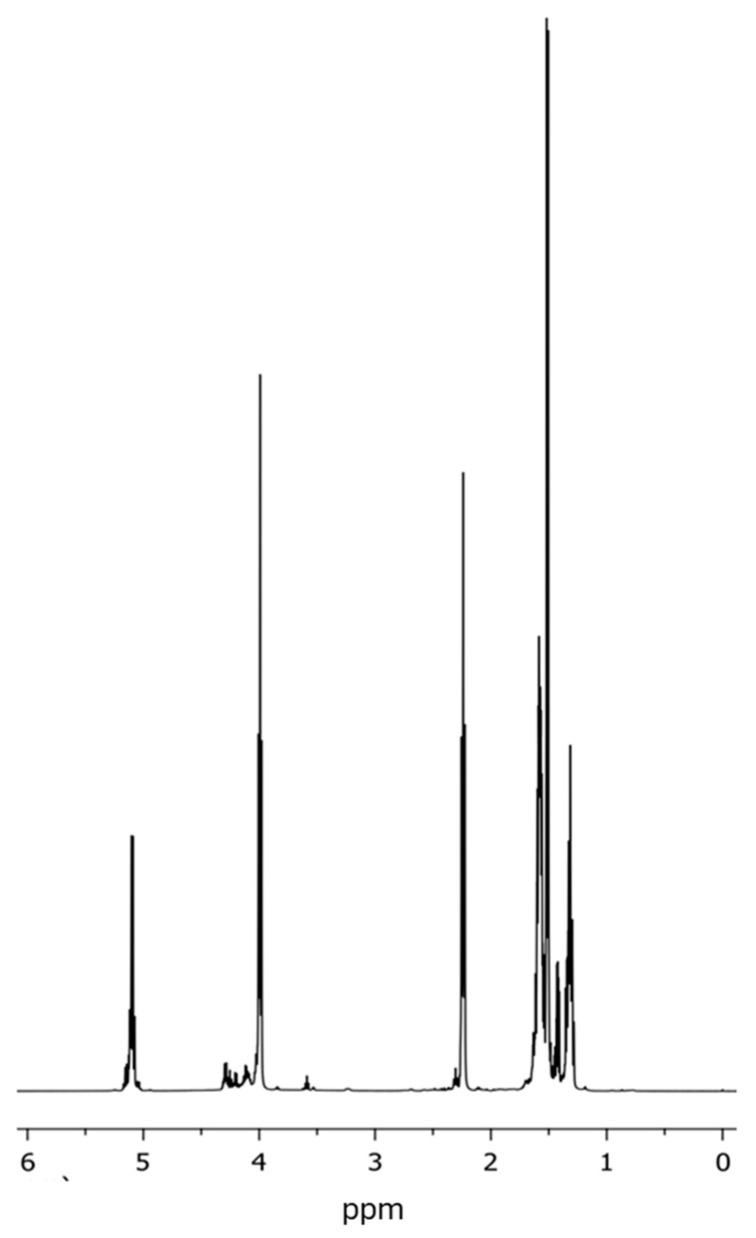
^1^H–NMR spectrum of the PLLA–PCL 2 copolymer.

The ^1^H–NMR spectrum of the PLLA–PCL 2 copolymer is shown in Figure 1. The PLLA/PCL ratio was determined through ^1^H NMR spectroscopy using the integral values for the signal assigned to the repeating groups in the synthesized copolyesters. In the case of PLLA, the methine group at *δ* = 5.2 ppm was used, whereas for PCL, the ε–methylene group at *δ* = 4.0 ppm was used.

The compositions in mol% of the synthesized PLLA–PCL copolymers are listed in Table 3. All copolymers are richer in PLLA. These results strongly suggest that a certain amount of PLLA end groups was not acylated during the treatment with OxCl. This means that not all PLLA chains possess terminal acyl chloride functionality. Therefore, in some PLLA chains, the hydroxyl terminal functionality remained and was able to react as the synthesis time was extended.

**Figure 2:**
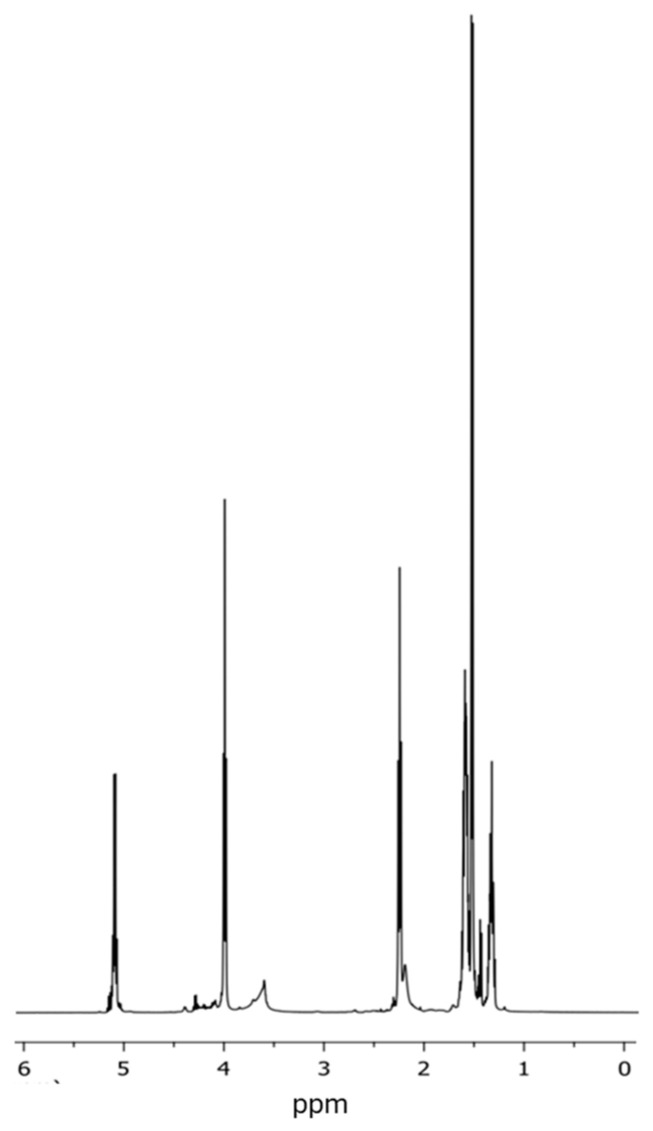
^1^H–NMR spectrum of the PLLA–PCL–PEG 3 terpolymer.

The ^1^H–NMR spectrum of the PLLA–PCL–PEG 3 terpolymer is shown in Figure 2. For the PLLA–PCL–PEG terpolymers, the integral for the signal at around 3.6 ppm (after deconvolution of the end group for PCL) was used to record the amount of PEG.

**Figure 3:**
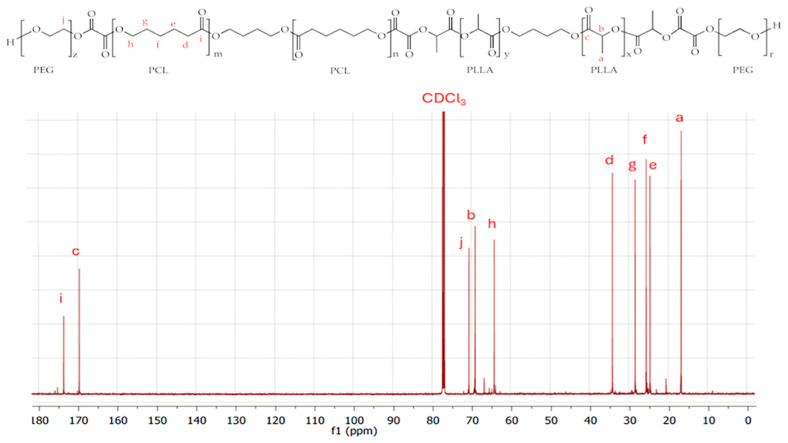
^13^C–NMR spectrum of the PLLA–PCL–PEG 2 terpolymer.

The ^13^C–NMR spectrum of the PLLA–PCL–PEG 2 terpolymer is shown in Figure 3. The signals expected for the three repeating units (PLA, PCL, and PEG) are present. The detection of all signals indicated that the PLLA–PCL–PEG terpolymers were successfully synthesized.

The compositions in mol% of the PLLA–PCL–PEG terpolymers are listed in Table 4. As expected, the composition of the prepared terpolymers was richer in the PLLA segments. In these PLLA–PCL–PEG terpolymers, the PEG blocks were included in low amounts (9.4–14 mol%). Nevertheless, the presence of PEG conferred a certain hydrophilic character to the hydrophobic PLLA–PCL copolymers, which was a desirable outcome for obtaining a nanocarrier sensitive to living matter. In this study, the PLLA–PCL–PEG 2 and PLLA–PCL–PEG 4 terpolymers are of special interest, owing to their high PCL content, as the hydrolysis of PCL has a strong influence on the drug-release behavior of a polymer prepared with PCL.

**Figure 4:**
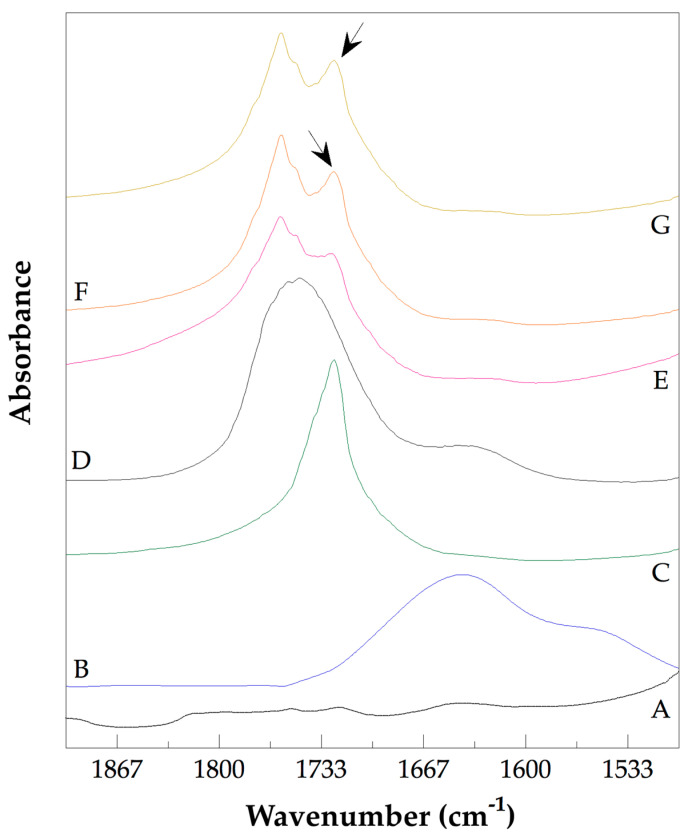
Partial IR spectra centered in the 1500 to 1900 cm^−1^ region of PEG (**A**), CNTs_po_ (**B**), PCL 4 (**C**), PLLA 1 (**D**), PLLA–PCL–PEG 2 (**E**), NC 2 (**F**), and NC 1 (**G**).

**Scheme 2:**
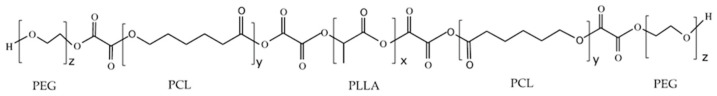
Chemical structure of the prepared PLLA–PCL–PEG terpolymers when water acts as an effective initiator.

A series of partial IR spectra of PEG, CNTs_po_, PCL 4, PLLA 1, PLLA–PCL–PEG 2, NC 2, and NC 1, presented in the region from 1500 to 1900 cm^−1^, are shown in Figure 4. The spectrum of pure PEG (Figure 4A) did not present a vibrational signal in the region analyzed. Contrarily, in the spectrum of CNTs_po_ (Figure 4B), a wide band at 1640 cm^−1^ was detected. This band was assigned to a stretching vibration of the C=C bond conjugated to the carbonyl group. Carbonyl functionality was introduced onto the CNT_po_ walls as a consequence of the oxidation process. Furthermore, the band extending to approximately 1730 cm^−1^ was attributed to the stretching vibrations of carboxyl groups, which self-associate to form dimers at approximately 1700 cm^−1^ and carboxyl-free groups at approximately 1720 cm^−1^. This helps resolve the wide band. The spectrum of PCL 4 (Figure 4C) exhibited a sharp peak at 1725 cm^−1^, which was caused by the stretching vibration of the ester carbonyl groups. For PLLA 1 (Figure 4D), the stretching vibration of the carbonyl groups of ester functionality produced a wide band at 1748 cm^−1^. In the spectrum of PLLA–PCL–PEG 2 (Figure 4E), a complex and wide band from 1650 cm^−1^ to ca. 1860 cm^−1^ was recorded. The vibrational movements of a high population of carbonyl groups were the cause of this wide band. At 1760 cm^−1^, the most intense contribution was detected. A weak shoulder of this band appeared as another band at 1750 cm^−1^. Subsequently, a well-resolved contribution at 1727 cm^−1^ was observed. Based on the IR spectral behavior of the pure homopolymers PCL 4 and PLLA 1 described above, two assignations were made. The weak band resolved at 1750 cm^−1^ was produced via the stretching vibration of the ester carbonyl groups of the segments of PLLA 1, whereas the band detected at 1727 cm^−1^ was caused by the stretching vibration of the ester carbonyl groups of the segments of PCL 4. Moreover, as shown in Scheme 1, the well-known chemical reaction between the hydroxyl groups (located on the terminal chains on PLLA, PCL, and PEG blocks) and the acyl chloride groups of the OxCl was expected to occur, forming the ester groups. As the vibrations of the carbonyl groups included in ester functionality resolve in the range of 1750 to 1715 cm^−1^ and the signal caused by the vibrations of dicarbonyl groups appears in the range of 1720 to 1705 cm^−1^, it is evident that the intense band detected at 1760 cm^−1^ originates from the vibrations of chemical groups different to ester carbonyl groups. Therefore, the band at 1760 cm^−1^ was assigned to the antisymmetric stretching vibration of the carbonyl groups of anhydride functionality. This assignation was confirmed by the fact that, in the range of 1500 to 500 cm^−1^ (region not shown), the most intense band was a double band resolved at 1212 and 1191 cm^−1^. This double band was caused by the symmetric and antisymmetric stretching vibrations of the carbonyl bonds of anhydride functionality. Anhydride functionality can be produced via the chemical reaction between the carboxyl and acyl chloride groups. Because wet BD was used in this synthesis, water moieties could act as effective alternative initiators on the homopolymerization of the PLLAs and PCLs prepared in steps 1 and 2 of the chemical procedure used in this study. A previous study reported that the homopolymerization of ε–CL initiated using water produced linear PCL chains with one carboxyl end group and one hydroxyl end group, whereas when dry BD was used as an initiator, the linear PCL synthesized contained two hydroxyl end groups [41]. Scheme 2 shows the chemical structure of the PLLA–PCL–PEG terpolymer obtained when water acted as an effective initiator of the series of prepared PLLAs and PCLs. In the IR spectra of NC 2 (Figure 4F) and NC 1 (Figure 4G), the spectral behavior was similar to that of the PLLA–PCL–PEG 2. Thus, in the IR spectra of both nanocomposites, three spectral contributions in 1760, 1750, and 1725 cm^−1^ were resolved. The bands detected at 1760 and 1750 cm^−1^ appeared at the same frequencies as those observed in the spectrum of PLLA–PCL–PEG 2 (Figure 4E) and were caused by the vibrations of the chemical groups already described. Instead, the band detected at 1725 cm^−1^ (marked on the spectra with an arrow) was resolved to a lower frequency and at a higher intensity than the one observed in the spectrum of PLLA–PCL–PEG 2. These changes could be attributed to the formation of intermolecular hydrogen bonds created between the carboxyl groups inserted on the walls of CNTs_po_ and the carbonyl groups of ester functionality of the PCL chains. The formation of hydrogen bonds in nanocomposites that were prepared with CNTs functionalized with carboxyl groups and a polymer containing carbonyl groups has been previously reported [42,43].

**Figure 5:**
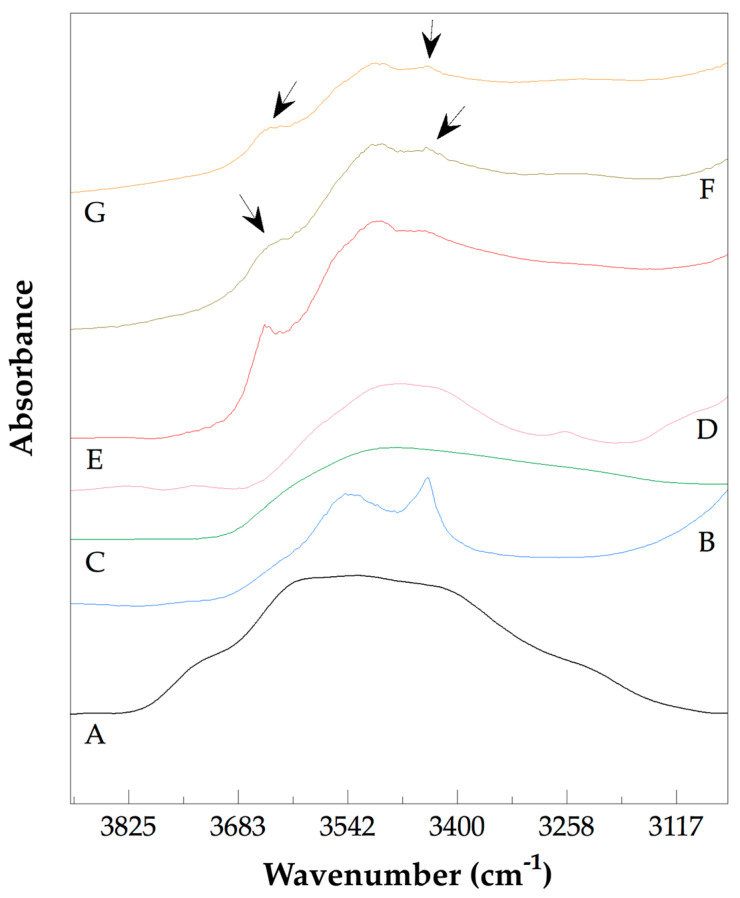
Partial IR spectra in the 3050-to-3900 cm^−1^ region for CNTs_po_ (**A**), PCL 4 (**B**), PLLA 1 (**C**), PEG (**D**), PLLA–PCL–PEG 2 (**E**), NC 2 (**F**), and NC 1 (**G**).

Figure 5 shows the partial spectra of CNTs_po_, PCL 4, PLLA 1, PEG, PLLA–PCL–PEG 2, NC 2, and NC 1 centered in the 3050-to-3900 cm^−1^ region. In the spectrum of CNTs_po_ (Figure 5A), a broad band at 3527 cm^−1^ due to the stretching vibration of the hydroxyl groups was detected. A similar broad band assigned to the stretching vibration of the hydroxyl groups on the FTIR spectrum of a sample of multiwalled carbon nanotubes (MWCNTs) has been reported elsewhere [44]. In the spectrum of PCL 4 (Figure 5B), a broad band with two peaks was detected. The first peak was sharp, and it was detected at 3438 cm^−1^, whereas the second peak was wide, and it appeared at 3536 cm^−1^. This broad band was due to the stretching vibration of the hydroxyl groups. For polymers that contain hydroxyl and carbonyl groups, the summation of the vibrations of several chemical bonds produces this band. From the lower to the higher frequencies, vibrations of different types of hydroxyl groups occurred: (i) self-associated hydroxyl groups forming dimers, trimers, etc.; (ii) hydrogen bonded to carbonyl groups; and (iii) free hydroxyl groups [45]. The vibrations of the free hydroxyl groups produced weak and sharp bands above 3615 cm^−1^ [46]. Therefore, we considered that the peak at 3438 cm^−1^ was caused by hydroxyl–hydroxyl self-associated movements, whereas the peak at 3536 cm^−1^ was due to hydroxyl–carbonyl vibrations. Concerning the PLLA 1 and PEG homopolymers, it is evident that their vibrational behaviors are similar. In the spectrum of PLLA (Figure 5C), only one band at 3479 cm^−1^ was detected, whereas in the spectrum of PEG (Figure 5D), also one band at 3470 cm^−1^ was observed. Both spectral contributions were assigned to the stretching vibration of the hydroxyl groups. Contrarily, in the spectrum of PLLA–PCL–PEG 2 (Figure 5E), three spectral contributions were detected. At 3649 cm^−1^, a weak sharp band assigned to vibrations of the free hydroxyl groups appeared. Moreover, a broad band at 3500 cm^−1^ with a shoulder at 3460 cm^−1^ was detected. The stretching vibration of the hydroxyl groups produced this broad band. In the spectra of NC 2 (Figure 5F) and NC 1 (Figure 5G), three bands were also detected. However, the band corresponding to vibrations of free hydroxyl groups (indicated by an arrow) was detected at lower frequencies, appearing in both spectra at 3637 cm^−1^. Meanwhile, the stretching vibration of the hydroxyl groups produced a broad band at 3502 cm^−1^ with shoulders at 3436 cm^−1^ (indicated by an arrow) (Figure 5F) and 3437 cm^−1^ (indicated by an arrow) (Figure 5G). The fact that these shoulders appeared at lower frequencies compared to the band detected at 3438 cm^−1^ in the spectrum of PCL 4 suggests that hydrogen bonds formed between the ester carbonyl groups of PCL 4 and the hydroxyl groups of the CNTs_po_ inserted in NC 2 and NC 1. These results, along with the results obtained from the analysis of the spectra shown in Figure 4E–G, suggest that the PCL 4 blocks develop a preferential orientation toward the CNTs_po_.

**Figure 6:**
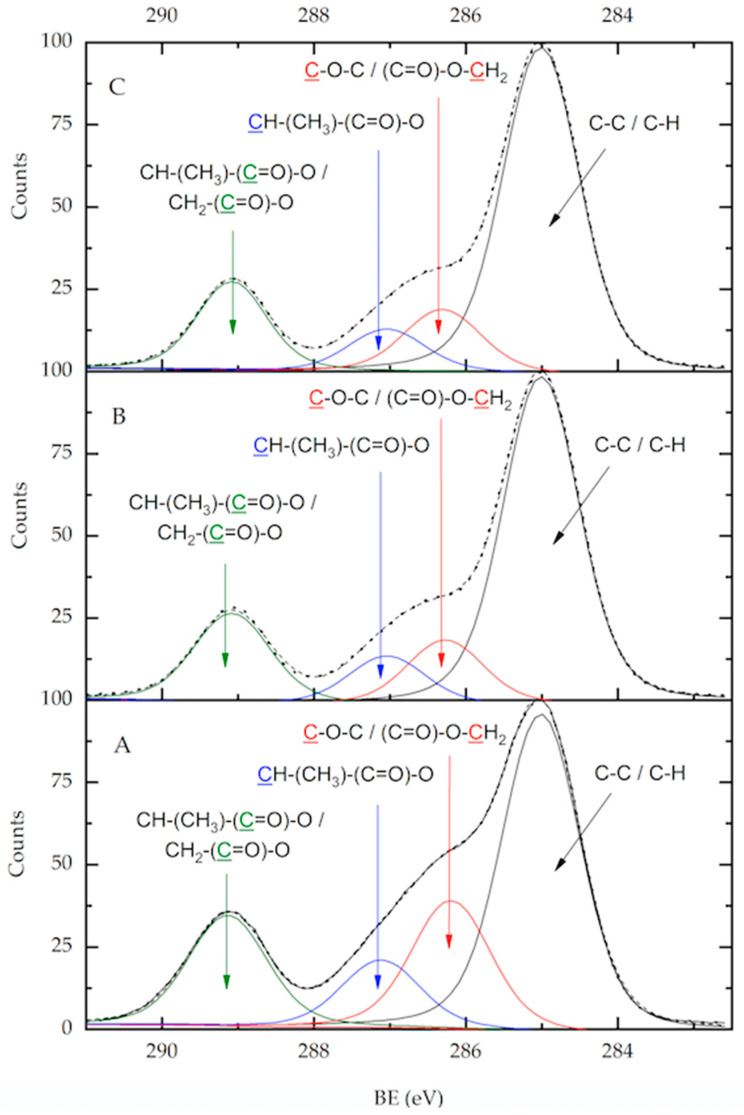
*C1s* core-level normalized spectra of PLLA–PCL–PEG 4 (**A**), NC 4 (**B**), and NC 3 (**C**).

**Scheme 3:**
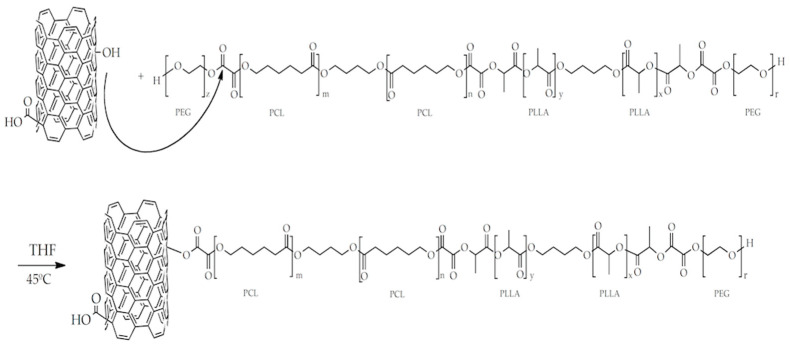
Grafting chemical reaction for preparing the PLLA–PCL–PEG/CNTs_po_ nanocomposites.

The *C1s* core-level normalized spectra of the PLLA–PCL–PEG 4 terpolymer, NC 4, and NC 3 are presented in Figure 6. The active background approach, as implemented in the Analyzer v. 1.33 software, was used to make the peaks fit [47]. The spectra were adjusted using the respective shift according to the C–C bond position at 285.0 eV. The *C1s* core-level normalized spectrum of the PLLA–PCL–PEG 4 terpolymer (Figure 6A) shows components at four positions: at 285.0 eV (C–C/C–H bonds) [48], 286.2 eV (C–O–C groups of PEG/–(C=O)–O–CH_2_– functionality of PCL) [49], 287.1 eV (–CH–(CH_3_)–(C=O)–O– functionality of PLLA) [49], and 289.1 eV ((–CH–(CH_3_)–(C=O)–O– functionality of the PLLA/–CH_2_–(C=O)–O–) groups of PCL) [49]. The calculated atomic concentrations of the mentioned groups were 36.4%, 14.7%, 7.8%, and 12.7%, respectively. In the *C1s* core-level normalized spectra of NC 4 and NC 3, components at four positions were also detected. The components and their calculated atomic concentrations detected in the spectrum of NC 4 (Figure 6B) were 285.0 eV (C–C/C–H bonds) [48] (46.0%), 286.3 eV (C–O–C groups of PEG/–(C=O)–O–CH_2_– functionality of PCL) [49] (9.1%), 287.0 eV (–CH–(CH_3_)–(C=O)–O– functionality of PLLA) [49] (6.8%), and 289.1 eV ((–CH–(CH_3_)–(C=O)–O– functionality of PLLA/–CH_2_–(C=O)–O–) groups of PCL) [49] (12.6%). For a similar analysis of the spectrum of NC 3 (Figure 6C), the results were 285.0 eV (C–C/C–H bonds) [48] (59.7%), 286.3 eV (C–O–C groups of PEG/–(C=O)–O–CH_2_– functionality of PCL) [49] (11.4%), 287.0 eV (–CH–(CH_3_)–(C=O)–O– functionality of PLLA) [49] (7.8%), and 289.1 eV ((–CH–(CH_3_)–(C=O)–O– functionality of PLLA/–CH_2_–(C=O)–O–) groups of PCL) [49] (14.9%). As previously reported, ester groups are sensitive nucleophilic substitution reactions [50]. The PLLA–PCL–PEG terpolymers are rich in ester groups. The nanocomposites were prepared in an aprotic solvent (THF). There are hydroxyl groups on the walls of the CNTs_po_. The hydroxyl group is nucleophilic and highly reactive. Moreover, in the chemical structure of the PLLA–PCL–PEG terpolymers, dicarbonyl groups exist. Because of the higher reactivity of the dicarbonyl groups compared to the carbonyl groups, it is possible that they are more sensitive to a chemical attack from the hydroxyl groups of the CNTs_po_. The decrease in the molar concentration of ester groups in the NC 3 and NC 4 nanocomposites (also detected in the *O1s* core-level normalized spectra of both nanocomposites (not shown)) compared to that in the PLLA–PCL–PEG 4 spectrum strongly suggests that a nucleophilic substitution reaction was performed. This implies that the chains of the PLLA–PCL–PEG 4 are grafted onto the walls of the CNTs_po_. Scheme 3 shows the aforementioned chemical reaction, in which the nucleophilic substitution on a carbonyl group of the dicarbonyl functionality is exemplified.

### 3.4. Thermal Analysis of the PLLA–PCL–PEG Terpolymers and PLLA–PCL–PEG/CNT_po_ Nanocomposites

**Figure 7:**
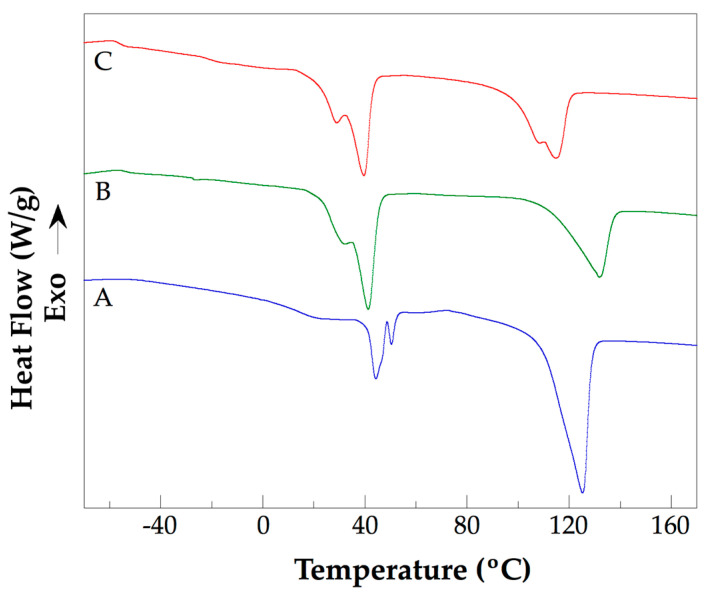
DSC thermograms of PLLA–PCL–PEG 4 (**A**), NC 3 (**B**), and NC 4 (**C**).

Figure 7 presents the DSC thermograms of PLLA–PCL–PEG 4, NC 3, and NC 4. The DSC results obtained from the analysis of the thermograms of PLLA–PCL–PEG 2, PLLA–PCL–PEG 4, NC 1, NC 2, NC 3, and NC 4 are summarized in Table 5. The Supplementary Material shows the DSC thermograms of the homopolymers PEG, PCL 4, and PLLA 4. The DSC thermal behavior of the pure homopolymers is described below. In the DSC thermogram of PEG, a glass-transition relaxation (T_gPEG_ = −13 °C (260.15K)) and a melting endotherm (T_mPEG_ = 61.0 °C (334.15 K), ΔH_mPEG_ = 60.8 J/g) were detected. In the DSC thermogram of PCL 4, a glass-transition temperature (T_gPCL 4_ = −59 °C (214.15 K)) and a melting endotherm (T_mPCL 4_ = 55.0 °C (328.15 K), ΔH_m PCL 4_ = 77.7 J/g) were detected. Furthermore, in the DSC thermogram of PLLA 1, a glass-transition temperature (T_gPLLA 1_ = 50 °C (323.15 K)), a crystallization exotherm (T_cPLLA 1_ = 111.6 °C (384.75 K), ΔH_cPLLA 1_ = 49.1 J/g), and a melting endotherm (T_mPLLA 1_ = 151.4 °C (424.55 K), ΔH_mPLLA 1_ = 42.6 J/g) were observed. Based on these results, the DSC thermogram of the PLLA–PCL–PEG 4 terpolymer was analyzed. In the DSC thermogram of PLLA–PCL–PEG 4 (Figure 7A), two glass-transition relaxations and two melting endotherms were detected. T_g1_ = −50 °C (223.15 K) was assigned to the relaxations in the amorphous phase of the PCL 4 chains, whereas T_g2_ = 14 °C (287.15 K) was assigned to domains rich in PEG chains. These thermal relaxations were detected at a higher temperature than those recorded in the thermograms of the pure homopolymers of PCL 4 and PEG, indicating that the crystalline phases detected in the thermogram of the terpolymer limited the relaxations in the amorphous phase. The first melting endotherm was resolved in a range of temperatures from 35 °C (308.15 K) to 55 °C (328.15 K) and exhibited two peaks. In this temperature range, relaxation in the amorphous phase of PLLA 1 chains, the melting of the PCL 4 chains, and the melting of the PEG chains occurred. The first peak, T_m1_ = 44.3 °C (317.45 K), is intense, while the second peak, T_m2_ = 50.4 °C (323.55 K), is weak. When a melted block copolymer is quenched below the melting temperature, microphase separation and crystallization occur simultaneously. Competition in the crystallization of the blocks is developed. Several studies have focused on the melting behavior of the PCL–PEG diblock copolymer. A study about PCL–PEG diblock copolymers with M_n_ = 20,000–30,000 prepared with PCL weight fractions ranging from 68 to 85% concluded that PCL blocks crystallize first, which induces an imperfect crystallization of PEG blocks [51,52]. Another study on the crystallization of synthesized PCL–PEG diblock copolymers with a PCL weight fraction of 34% to 67% concluded that PCL chains crystallize first. After this, the crystallization of PEG chains occurs [53]. Xu et al. studied the crystallization of PCL–PEG copolymers with M_n,PEG_ = 5000 and concluded that PCL blocks disfavor the crystallization of PEG blocks, and vice versa [54]. This explains the small value of ΔH_m1_ (shown in Table 5) for both PLLA–PCL–PEG 4 and PLLA–PCL–PEG 2. Furthermore, in another study of a PEG–PCL diblock copolymer prepared with PEG (M_n_ = 5000) and PCL (M_n_ = 9200), two melting peaks were observed in the melting endotherm detected through DSC, which were assigned to the melting of the PEG (first peak) domains and melting of the PCL (second peak) domains. XRD measurements support this analysis [55]. Similar results were recorded in a study of segmented PCL/PEG-based poly(urethane urea) copolymers prepared with a PCL/PEG weight ratio of 80/20 and M_w_ of 67000. On the melting endotherm, two melting peaks were detected. The first peak was caused by the melting of the PEG domains and the second peak by the melting of the PCL domains [56]. Therefore, it is reasonable to consider that, in the DSC endotherm of PLLA–PCL–PEG 4, the first peak (T_m1_ = 44.3 °C (317.45 K)) was caused by the melting of the PEG domains, and the second peak (T_m2_ = 50.4 °C (323.55 K)) was due to the melting of the PCL 4 domains. In some cases, in the endotherm of the PCL–PEG copolymers, only one T_m_ has been detected, as reported elsewhere [57]. As shown in Table 5, for PLLA–PCL–PEG 2, only one melting temperature (T_m1_ = 44.3 °C (317.45 K)) was detected, indicating the simultaneous crystallization of PCL and PEG blocks. Additionally, the second endotherm in the DSC thermogram of the PLLA–PCL–PEG 4 terpolymer was detected in the temperature range from approximately 100 °C (373.15 K) to 135 °C (408.15 K), in which T_m3_ = 125.4 °C (398.55 K) was detected, attributed to the melting of the PLLA 1 domains. Evidently, this melting temperature was detected to a lower temperature than the melting temperature of the pure PLLA 1. This means that the complex morphology of the PLLA–PCL–PEG 4 terpolymer has a strong influence on the melting of the PLLA 1 domains.

Regarding the thermal behavior of the nanocomposites, although two glass-transition relaxations and two melting endotherms were observed in their DSC thermograms (Figure 7B for NC 3 and Figure 7C for NC 4), significant changes were detected compared to those observed in the thermograms of their pure polymer matrices. Thus, (i) for all nanocomposites, T_g1_ due to the glass transition of the PCL 4 chains was detected at lower temperatures than those observed for their pure polymeric matrix; (ii) for NC 2 and NC 4 (both prepared with 0.5 wt.% CNTs_po_), T_g2_ caused by the glass-transition relaxation of the PEG chains was detected at a lower temperature than that of pure PEG. Contrarily, for NC 1 and NC 3 (both prepared with 1.0 wt.% CNTs_po_), T_g2_ was detected at a higher temperature. The increase in the value of the glass-transition temperature of nanocomposites prepared with CNTs has been explained as a consequence of the reduction in the mobility of the polymer chains. This reduction is induced via the physical and chemical interactions formed between the chemical groups inserted on the walls of the CNTs_po_ and the chemical groups of the polymer chains of the polymeric matrices of the studied nanocomposites. Similar results for the nanocomposites of PMMA and amino-functionalized CNTs have been reported elsewhere [58]. Our results indicate that the reduction in the mobility of polymer chains depends on the amount of CNTs_po_ used in the preparation of the studied nanocomposites. Contrarily, the decrease in the glass-transition temperature has been explained as a consequence of the high dispersion of CNTs and the formation of a nanoscale thin film of confined polymer between CNTs, as reported in a study of nanocomposites prepared with polycarbonate (as the polymer matrix) and functionalized MWCNTs previously published [59]. The small amount of CNTs_po_ used to prepare the NC 2 and NC 4 favors achieving a high dispersion of CNTs_po_. Moreover, the presence of CNTs_po_ in the studied nanocomposites has a different influence on the melting of domains rich in PEG/PCL 4 chains or domains rich in PLLA 1 chains. Thus, a clear change is observed in the first endotherm of the DSC thermograms of all the prepared nanocomposites. Unlike the DSC thermogram of the PLLA–PCL–PEG 4 terpolymer, where the most intense peak is the first, corresponding to the melting of PEG chains, for the nanocomposites NC 3 and NC 4, the most intense peak is the second. This second peak is produced through the melting of the PCL chains. Furthermore, the ΔH_m1_ of all the nanocomposites is larger than those of their polymer matrices (PLLA–PCL–PEG 2 and PLLA–PCL–PEG 4). These results indicate that CNTs_po_ act as nucleation sites of the PCL 4 chains. The role of CNTs (functionalized with the hydroxyl and carboxyl groups) as nucleating agents in nanocomposites prepared with a semicrystalline polymer has been previously reported [60]. Contrarily, for the second endotherm, the ΔH_m2_ (associated with the melting of PLLA 1 chains) of all the nanocomposites decreased compared to the second endotherm observed in the DSC thermograms of the pure polymer matrices. In this case, CNTs_po_ act as antinucleating agents. The thermal behavior observed in the melting of the prepared nanocomposites strongly suggests that the PCL 4 chains are preferentially oriented to CNTs_po_.

### 3.5. Analysis of In Vitro Methotrexate Release Profiles from PLLA–PCL–PEG Terpolymers and PLLA–PCL–PEG/CNT_po_ Nanocomposites

**Figure 8:**
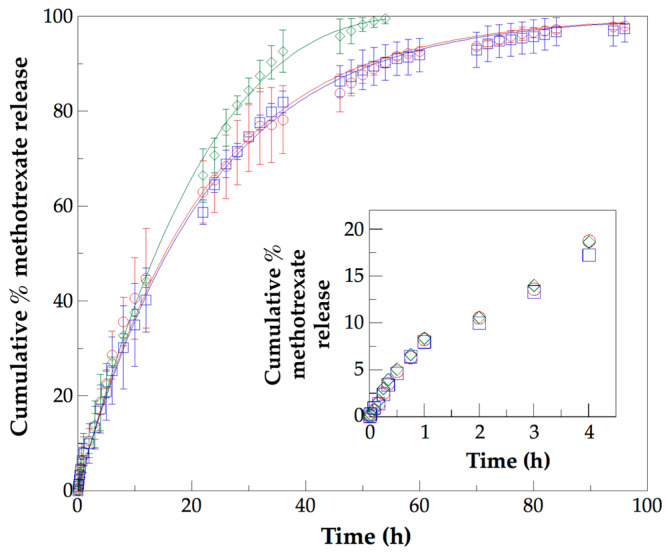
In vitro methotrexate release profiles for PLLA–PCL–PEG 2 (◇), NC 1 (◯), and NC 2 (☐). An inset shows the methotrexate release data without error bars until 4 h.

**Figure 9:**
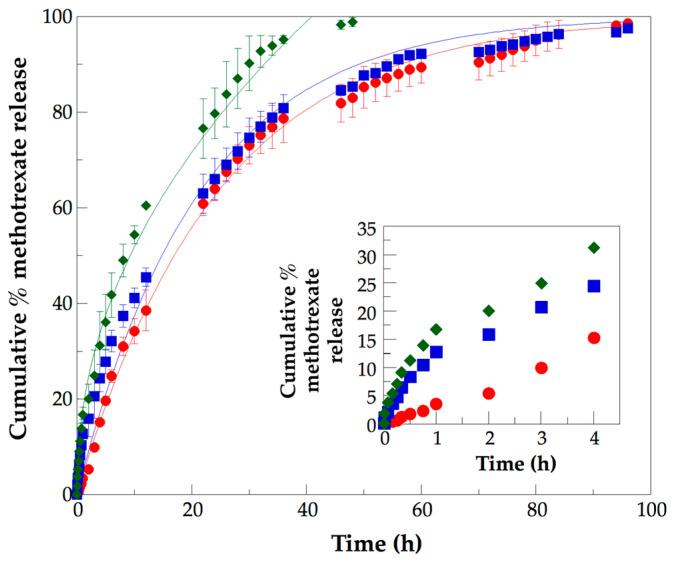
In vitro methotrexate release patterns for PLLA–PCL–PEG 4 (◆), NC 3 (●), and NC 4 (■). An inset shows the methotrexate release data without error bars until 4 h.

Figure 8 presents the in vitro methotrexate release patterns for the PLLA–PCL–PEG 2 terpolymer and NC 1 and NC 2, whereas Figure 9 shows the release profiles for the PLLA–PCL–PEG 4 terpolymer and NC 3 and NC 4. In both figures, the error bars of all the recovered data are included. An inset is included in which the methotrexate release data recorded until 4 h are shown. During the release test, the tablets of the terpolymers and the nanocomposites underwent erosion. The erosion was faster for the PLLA–PCL–PEG 2 and PLLA–PCL–PEG 4 terpolymers compared to the nanocomposites prepared with them. Moreover, the release from the terpolymers was faster than that from the nanocomposites. Indeed, the methotrexate release rates from PLLA–PCL–PEG 2 and PLLA–PCL–PEG 4 were 44.1% and 60.4% at 12 h, respectively. The highest release was obtained at 54 h for PLLA–PCL–PEG 2 (99.5%) and at 48 h for PLLA–PCL–PEG 4 (98.8%). Contrarily, the methotrexate release rates for NC 1, NC 2, NC 3, and NC 4 were 41.8%, 41.0%, 38.8%, and 43.1% at 12 h, respectively. Moreover, the highest release rates for NC 1, NC 2, NC 3, and NC 4 were 98.7%, 98.5%, 98.0%, and 98.8% at 96 h, respectively. Evidently, the release evolution of all the studied terpolymers and nanocomposites follows a biphasic cumulative release behavior characterized by a “burst release” at the initial times and a plateau (“power–law phase”), which is smaller for PLLA–PCL–PEG 2 and PLLA–PCL–PEG 4 than for the nanocomposites. The “burst release” for the nanocomposites in the first moments was attenuated, as can be observed in the insets of Figure 8 and Figure 9. This effect was more evident for NC 3 and NC 4, which were prepared with a polymer matrix of PLLA–PCL–PEG 4. The fact that this terpolymer has a lower content of PEG than the PLLA–PCL–PEG 2 terpolymer indicates that it is more hydrophobic, which, combined with the presence of CNTs_po_, favors a slower methotrexate release.

Five models were used to fit the methotrexate release data. These models were Higuchi (1), Korsmeyer–Peppas (2), Korsmeyer–Peppas extended (3), first-order (4), and Hixson–Crowell (5). In all cases, *F* denotes the percentage of drug released, which is determined from $M_{t}/M_{\infty }$, and the fraction of drug release at time *t*, where $M_{t}$ denotes the amount of drug released at time *t*, and $M_{\infty }$ denotes the amount of drug loaded:

(1) $$F=k_{H}\;t^{\frac{1}{2}}$$

where $k_{H}$ denotes the kinetic rate constant of the Higuchi model, which depends on the variables of the release system, and *t* denotes the time. Higuchi’s model is based on Fick’s second law [61].

(2) $$F=k_{KP}\;t^{n}$$

where $k_{KP}$ denotes the kinetic rate constant of the Korsmeyer–Peppas model, and *n* is the release exponent. This exponent characterizes the release mechanism from polymer matrices, which can have different geometries. The Korsmeyer–Peppas model was proposed to assess the released drugs from a polymer matrix [62].

(3) $$F=k_{KP}^{\prime }\;t^{n}+b$$

where $k_{KP}^{\prime }$ denotes the kinetic rate constant of the Korsmeyer–Peppas extended model, and *b* denotes the “burst effect.” This model is an extension of the Korsmeyer–Peppas model, and it was proposed to evaluate the “burst effect” [63].

(4) $$F=100\;\left(1-e^{-k_{1}\;t}\right)$$

where $k_{1}$ denotes the first-order release constant [64]. Using Fick’s first law, it is possible to obtain a relationship among *k*_1_ and the diffusion constant in the release medium. A wide variety of systems follow this model.

(5) $$F=100\;\left[1-{\left(1-k_{HC}\;t\right)}^{3}\right]$$

where $k_{HC}$ denotes the release constant of the Hixson–Crowell model [65]. This model applies to tablets, and it considers the dissolution carried out in planes parallel to the surface of the tablet.

The DDSolver software was used to fit the experimental data to the drug-release kinetic models [66]. Among the statistical criteria provided by DDSolver to evaluate the goodness of fit of a model, the coefficient of determination (R^2^), mean square error (MSE), and model selection criterion (MSC) were used in this study. In the comparison of different models, the most appropriate model is the one with the largest MSC. A good fit is achieved when an MSC value larger than 2 or 3 is determined [67]. Table 6 shows the kinetic parameters for methotrexate release from tablets of PLLA–PCL–PEG 2 terpolymer and NC 1 and NC 2 nanocomposites. In contrast, Table 7 presents the kinetic parameters obtained for release tests realized with tablets of PLLA–PCL–PEG 4 terpolymer and NC 3 and NC 4 nanocomposites.

For NC 1, NC 2, NC 3, and NC 4, a comparison of the results shown in Table 6 and Table 7 indicates that the first-order model fit the experimental data with the best accuracy. This statement is based on the fact that the R^2^ values were generally close to the unity, the MSE values were minimal, and the MSC values were maximal. Contrarily, when these three criteria are taken into account, for the PLLA–PCL–PEG 2 and PLLA–PCL–PEG 4 terpolymers, the best models were Hixson–Crowell and Korsmeyer–Peppas, respectively. In Figure 8 and Figure 9, the solid lines traced over the experimental data of each type of nanocomposite or terpolymer correspond to the fit of the best model mentioned above.

It is important to analyze the result obtained from the Korsmeyer–Peppas model, as its exponent *n* provides information regarding the drug-release mechanism. The values of *n* for the nanocomposites were 0.44 (NC 1), 0.45 (NC 2), 0.47 (NC 3), and 0.40 (NC 4). Therefore, Fickian diffusion drives the methotrexate release, specifically for a planar (thin-film) matrix (NC 3), a cylinder-shaped matrix (NC 1 and NC 2), and a sphere-shaped matrix (NC 4). For the last nanocomposite (NC 4), the value of *n* was minor compared to the typical value of *n* (0.43) reported elsewhere [68]. Nevertheless, in a similar case, an analogous classification was reported previously [69]. For the PLLA–PCL–PEG 4 terpolymer, the *n* value was 0.47, indicating diffusion from a planar matrix. However, for PLLA–PCL–PEG 2, the *n* value was 0.59, indicating an anomalous transport for a planar matrix. Moreover, the analysis through the Korsmeyer–Peppas extended model determined that the *n* value of PLLA–PCL–PEG 2 terpolymer was 0.77, whereas for NC 1 and NC 2, the values were 0.78 and 0.76, respectively, indicating an anomalous transport for a planar matrix. For all these materials, the value of *b* was 0.12, indicating that the presence of CNTs_po_ has a small influence on the “burst effect.” Contrarily, for PLLA–PCL–PEG 4 terpolymer, the value of *n* was 0.61, and the value of *b* was 0.89, while for NC 3 and NC 4, the values of *n* were 0.86 and 0.56, respectively, but for both nanocomposites, the values of *b* were negative. As negative values do not have a physical meaning, these results indicate a poor fit.

The fact that methotrexate is poorly water-soluble (approximately 65 μg/mL) [70] favors its distribution on the PCL chains due to the hydrophobic nature of this biopolymer [14]. In this sense, the lower values of ΔH_m1_ for the prepared terpolymers and nanocomposites compared to the ΔH_m PCL 4_ of pure PCL 4 indicate a reduction in crystallinity, which favors the faster hydrolysis of the PCL domains [71] induced via an increment in the mobility of the PCL chains [72]. This affects the release of methotrexate. Although the “burst effect” was not substantially reduced for the studied nanocomposites, it is evident that, in the release tests performed with the nanocomposites, the methotrexate release time was extended, which can be a positive factor in the consideration of their possible use.

## 4. Conclusions

PLLA–PCL–PEG/CNT_po_ nanocomposites were prepared and characterized through DSC, as well as FTIR, and XPS spectroscopies, to evaluate their ability to act as nanocarriers of methotrexate. CNTs were prepared through CVD, purified, and chemically functionalized to achieve partial oxidation. The content of the carboxyl groups was higher than that of the hydroxyl groups in the CNTs_po_. Wet BD was used as an initiator to prepare a series of linear PLLAs and PCL through ROP. The water in the wet BD acted as an effective co-initiator in the synthesis of these homopolymers. PLLA–PCL copolymers and PLLA–PCL–PEG segmented terpolymers were synthesized using OxCl as an effective linker agent. These terpolymers exhibit strong hydrophobic characteristics due to the high content of PLLA and PCL blocks. The evidence recorded using FTIR strongly suggests that the carboxyl and hydroxyl groups of the CNTs_po_ form hydrogen bonding with the ester carbonyl groups of the PCL chains. Moreover, the CNTs_po_ act as nucleating agents of the PCL chains. This suggests that the PCL chains are preferentially oriented to CNTs_po_. The XPS measurements indicated that the PLLA–PCL–PEG terpolymers chains were grafted to CNTs_po_. For both PLLA–PCL–PEG terpolymers and PLLA–PCL–PEG/CNT_po_ nanocomposites, a “burst release” of methotrexate was detected. The “burst release” was attenuated but was not eliminated for the nanocomposites. The in vitro release behavior was analyzed using five models. For all the nanocomposites, the best fit was obtained with the first-order model, whereas for the PLLA–PCL–PEG 2 and PLLA–PCL–PEG 4 terpolymers, the best fit was determined with the Hixson–Crowell and Korsmeyer–Peppas models, respectively. The analysis conducted with the Korsmeyer–Peppas model revealed that, in general, for both the nanocomposites and terpolymers studied, Fickian diffusion drives the methotrexate release.

## Figures and Tables

**Table 1:** Identification legends for the PLLA–PCL–PEG terpolymers, the PLLA–PCL–PEG/CNTs_po_ nanocomposites, and the content of CNTs_po_ (wt.%) in each nanocomposite.

Legend Assigned to the Prepared PLLA–PCL–PEG/CNTs_po_ Nanocomposites	Name of the PLLA–PCL–PEG Terpolymer Used as the Polymer Matrix of a Nanocomposite	Content of CNTs_po_ (wt.%)
NC 1	PLLA–PCL–PEG 2	1.0
NC 2	PLLA–PCL–PEG 2	0.5
NC 3	PLLA–PCL–PEG 4	1.0
NC 4	PLLA–PCL–PEG 4	0.5

**Table 2:** GPC results and yield of the homopolymerization of L–LA and ε–CL; both polymer precursors are denominated here as M.

Homopolymer	Molar Ratio of [M]/[Wet BD]	M_w_ (g/mol)	M_w_/M_n_	Yield (%)
PLLA 1	30/1	24,670	1.07	90
PLLA 2	40/1	31,110	1.08	92
PLLA 3	50/1	34,250	1.09	95
PLLA 4	60/1	34,800	1.07	90
PCL 1	30/1	10,280	1.07	90
PCL 2	40/1	25,650	1.10	90
PCL 3	50/1	32,330	1.16	93
PCL 4	60/1	35,370	1.11	91

**Table 3:** Compositions in mol% of PLLA–PCL copolymers.

Copolymer	PLLA	PCL
PLLA–PCL 1	63.0	37.0
PLLA–PCL 2	52.8	47.2
PLLA–PCL 3	62.1	37.9
PLLA–PCL 4	54.7	45.3

**Table 4:** Compositions in mol% of PLLA–PCL–PEG terpolymers.

Terpolymer	PLLA	PCL	PEG
PLLA–PCL–PEG 1	68.2	22.4	9.4
PLLA–PCL–PEG 2	46.0	40.0	14.0
PLLA–PCL–PEG 3	51.0	38.4	10.6
PLLA–PCL–PEG 4	48.0	43.0	9.0

**Table 5:** Results of the analysis of the DSC thermograms of the PLLA–PCL–PEG terpolymers and PLLA–PCL–PEG/CNTs_po_ nanocomposites used in methotrexate-release tests.

Sample	T_g1_ (K)	T_g2_ (K)	T_m1_ (K)	T_m2_ (K)	ΔH_m1_ J/g	T_m3_ (K)	ΔH_m2_ J/g
PLLA–PCL–PEG 2	221.15	287.15	318.45	–	8.1	398.85	35.9
PLLA–PCL–PEG 4	223.15	285.15	317.45	323.55	8.0	398.55	36.6
NC 1	216.15	291.15	313.95	319.85	30.2	413.65	29.6
NC 2	218.15	258.15	312.65	319.45	33.6	413.35	28.5
NC 3	219.15	290.15	305.55	314.45	26.4	405.05	19.9
NC 4	217.15	252.15	302.15	312.75	23.3	387.95	20.5

**Table 6:** Kinetic parameters for methotrexate release from tablets of PLLA–PCL–PEG 2 terpolymer and NC 1 and NC 2 nanocomposites determined with Higuchi, Korsmeyer–Peppas, Korsmeyer–Peppas extended, first-order, and Hixson–Crowell models.

Models		Tested Materials		
		PLLA–PCL–PEG 2	NC 1	NC 2
Higuchi	$k_{H}\;\left(h^{-\frac{1}{2}}\right)$	14.12 ± 0.36	11.56 ± 0.29	11.51 ± 0.24
	R^2^	0.975 ± 0.013	0.963 ± 0.024	0.960 ± 0.002
	MSE	39.95 ± 23.93	53.16 ± 33.81	59.26 ± 6.58
	MSC	3.67 ± 0.54	3.31 ± 0.70	3.11 ± 0.05
Korsmeyer–Peppas	$k_{KP}\;\left(h^{-n}\right)$	10.34 ± 0.71	14.87 ± 3.61	14.18 ± 2.89
	*n*	0.59 ± 0.03	0.44 ± 0.05	0.45 ± 0.06
	R^2^	0.984 ± 0.008	0.972 ± 0.012	0.966 ± 0.008
	MSE	26.57 ± 16.16	41.72 ± 17.77	51.47 ± 19.53
	MSC	4.02 ± 0.53	3.46 ± 0.46	3.26 ± 0.23
Korsmeyer–Peppas extended^1^	$k_{KP}^{\prime }\;\left(h^{-n}\right)$	6.92 ± 3.69	6.64 ± 0.49	6.47 ± 3.36
	*n*	0.77 ± 0.24	0.78 ± 0.05	0.76 ± 0.14
	*b*	0.12 ± 0.69	0.12 ± 0.37	0.12 ± 0.17
	R^2^	0.995 ± 0.003	0.993 ± 0.004	0.996 ± 0.002
	MSE	1.19 ± 0.85	1.68 ± 0.23	0.88 ± 0.73
	MSC	4.99 ± 0.70	4.60 ± 0.54	5.17 ± 0.55
First-order	$k_{1}\left(h^{-1}\right)$	0.06 ± 0.003	0.05 ± 0.008	0.04 ± 0.001
	R^2^	0.991 ± 0.001	0.996 ± 0.003	0.993 ± 0.005
	MSE	13.88 ± 3.78	5.85 ± 3.91	10.01 ± 5.32
	MSC	4.61 ± 0.16	5.53 ± 0.74	4.96 ± 0.72
Hixson–Crowell	$k_{HC}\left(h^{-\frac{1}{3}}\right)$	0.015 ± 0.001	0.012 ± 0.002	0.012 ± 0.001
	R^2^	0.995 ± 0.004	0.985 ± 0.010	0.983 ± 0.020
	MSE	6.47 ± 5.28	21.18 ± 13.68	21.60 ± 25.91
	MSC	5.56 ± 1.04	4.23 ± 0.71	4.75 ± 1.93

^1^ To determine the burst effect, the portion of the release curve where *F* < 44.8% was analyzed.

**Table 7:** Kinetic parameters for methotrexate release from tablets of PLLA–PCL–PEG 4 terpolymer and NC 3 and NC 4 nanocomposites determined with Higuchi, Korsmeyer–Peppas, Korsmeyer–Peppas extended, first-order, and Hixson–Crowell models.

Models		Tested Materials		
		PLLA–PCL–PEG 4	NC 3	NC 4
Higuchi	$k_{H}\;\left(h^{-\frac{1}{2}}\right)$	15.86 ± 0.35	11.26 ± 0.38	11.56 ± 0.17
	R^2^	0.985 ± 0.002	0.966 ± 0.006	0.961 ± 0.015
	MSE	19.58 ± 4.40	50.50 ± 13.51	51.48 ± 17.69
	MSC	4.10 ± 0.11	3.29 ± 0.18	3.16 ± 0.38
Korsmeyer–Peppas	$k_{KP}\;\left(h^{-n}\right)$	17.65 ± 1.93	12.71 ± 0.14	17.20 ± 2.33
	*n*	0.47 ± 0.04	0.47 ± 0.01	0.40 ± 0.03
	R^2^	0.989 ± 0.006	0.967 ± 0.007	0.979 ± 0.002
	MSE	16.33 ± 10.25	49.82 ± 14.44	28.39 ± 2.04
	MSC	4.34 ± 0.56	3.28 ± 0.20	3.70 ± 0.10
Korsmeyer–Peppas extended^1^	$k_{KP}^{\prime }\;\left(h^{-n}\right)$	13.75 ± 5.67	4.88 ± 0.33	11.83 ± 3.21
	*n*	0.61 ± 0.14	0.86 ± 0.06	0.56 ± 0.09
	*b*	0.89 ± 2.83	– 0.86 ± 0.18	– 0.38 ± 0.51
	R^2^	0.993 ± 0.005	0.988 ± 0.003	0.997 ± 0.001
	MSE	2.66 ± 1.66	2.43 ± 0.15	0.65 ± 0.11
	MSC	4.76 ± 0.79	4.05 ± 0.23	5.54 ± 0.23
First-order	$k_{1}\left(h^{-1}\right)$	0.08 ± 0.001	0.04 ± 0.004	0.05 ± 0.005
	R^2^	0.981 ± 0.015	0.996 ± 0.003	0.987 ± 0.006
	MSE	25.01 ± 17.65	5.55 ± 3.84	16.28 ± 7.78
	MSC	3.98 ± 0.89	5.61 ± 0.85	4.34 ± 0.53
Hixson–Crowell	$k_{HC}$ $\left(h^{-\frac{1}{3}}\right)$	0.02 ± 0.001	0.01 ± 0.001	0.01 ± 0.001
	R^2^	0.964 ± 0.032	0.984 ± 0.011	0.970 ± 0.010
	MSE	46.05 ± 37.69	21.98 ± 14.72	39.53 ± 11.87
	MSC	3.43 ± 1.05	4.23 ± 0.82	3.41 ± 0.34

^1^ The burst effect was evaluated through an analysis of the portion of the release curve in which F < 60.5%.

## Data Availability

The data presented in this study are available upon request from the corresponding author.

## Supplementary Materials

The following supporting information can be downloaded at https://www.mdpi.com/article/10.3390/polym16182580/s1: Figure S1: DSC thermograms of pure PEG (A), PCL 4 (B), and PLLA 1 (C).
