# Supplementary material for: Synthesis of Poly(L–lactide)–poly(ε–caprolactone)–poly(ethylene glycol) Terpolymer Grafted onto Partially Oxidized Carbon Nanotube Nanocomposites for Drug Delivery
Source: Polymers (Basel). 2024 Sep 12;16(18):2580. doi: 10.3390/polym16182580 (PMC11435386; doi:10.3390/polym16182580)
Supplement: Supplementary file 1 [file polymers-16-02580-s001.zip › polymers-3188012-supplementary.pdf]

Synthesis of Poly(L–lactide)–poly(ε–caprolactone)–poly(ethylene glycol) Terpolymer Grafted onto Partially Oxidized Carbon Nanotube Nanocomposites for Drug Delivery

Karla J. González-Iñiguez, Edgar B. Figueroa-Ochoa, Antonio Martínez-Richa, Leonardo R. Cajero-Zul and Sergio M. Nuño-Donlucas

Supplementary Material

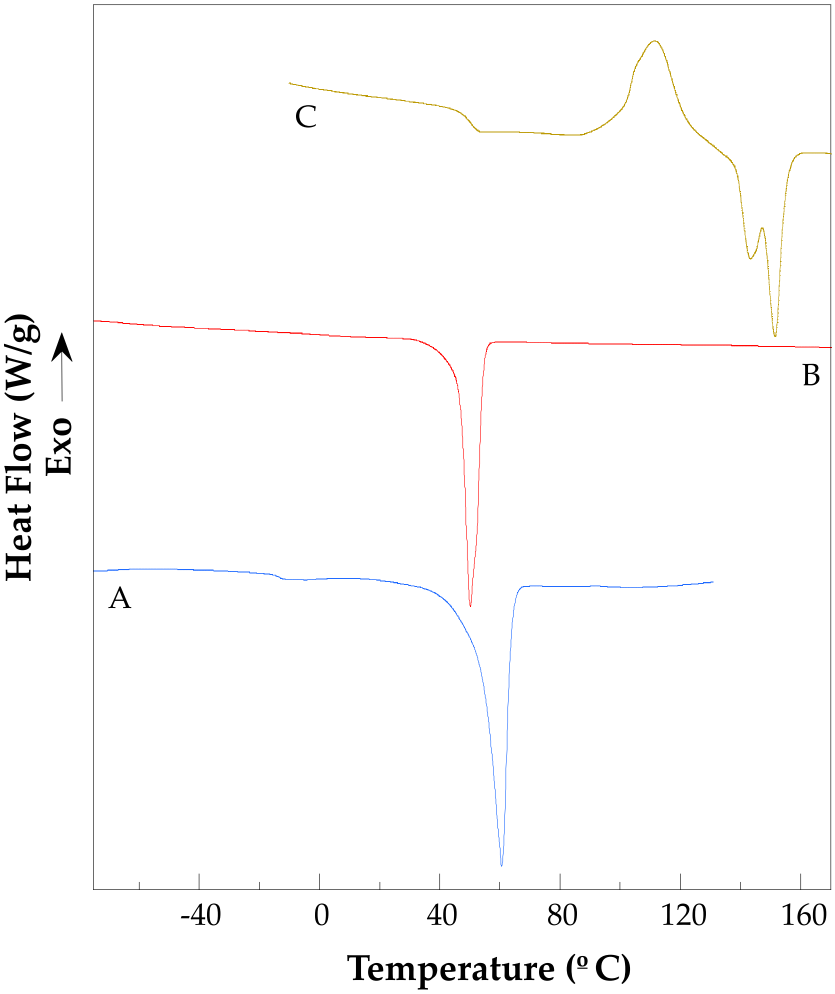

Figure S1. DSC thermograms of pure PEG (A), PCL 4 (B), and PLLA 1 (C).
